# Modelling transport of inhibiting and activating signals and their combined effects on floral induction: application to apple tree

**DOI:** 10.1038/s41598-020-69861-8

**Published:** 2020-08-04

**Authors:** Fares Belhassine, Damien Fumey, Jérôme Chopard, Christophe Pradal, Sébastien Martinez, Evelyne Costes, Benoît Pallas

**Affiliations:** 1 AGAP, Univ Montpellier, INRAE, CIRAD, Montpellier SupAgro, Montpellier, France; 2 ITK, Clapiers, France; 3 CIRAD, UMR AGAP, Montpellier, France

**Keywords:** Shoot apical meristem, Flowering, Computational models, Plant signalling

## Abstract

Floral induction (FI) in shoot apical meristems (SAM) is assumed to be triggered by antagonistic endogenous signals. In fruit trees, FI occurs in some SAM only and is determined by activating and inhibiting signals originating from leaves and fruit, respectively. We developed a model (SigFlow) to quantify on 3D structures the combined impact of such signals and distances at which they act on SAM. Signal transport was simulated considering a signal ‘attenuation’ parameter, whereas SAM fate was determined by probability functions depending on signal quantities. Model behaviour was assessed on simple structures before being calibrated and validated on a unique experimental dataset of 3D digitized apple trees with contrasted crop loads and subjected to leaf and fruit removal at different scales of tree organization. Model parameter estimations and comparisons of two signal combination functions led us to formulate new assumptions on the mechanisms involved: (i) the activating signal could be transported at shorter distances than the inhibiting one (roughly 50 cm vs 1 m) (ii) both signals jointly act to determine FI with SAM being more sensitive to inhibiting signal than activating one. Finally, the genericity of the model is promising to further understand the physiological and architectural determinisms of FI in plants.

## Introduction

Timing and intensity of floral induction (FI) are key processes in plants that strongly determine their reproductive ability. Most of the fruit trees have the particularity to induce floral transition in meristems the year before flowering^1^ and in part of the meristems, only^2^. FI usually takes place in spring when fruit growth also occurs, which can in turn affect vegetative growth and the proportion of meristems that are floral induced. The most known illustrations of this potential conflict between growth and FI are biennial bearing and masting that are associated with one year of high flowering intensity following one or many years of low fruit load^3^. Moreover, within a tree canopy, meristems are subjected to contrasted conditions due to variations in microclimate, shoot polymorphism or fruit location^4^. Such variations in local conditions can affect flowering as shown by the positive correlation between shoot length and FI^5,6^, or by the decreasing proportion of FI in fruiting tree parts^7^. Several hypotheses have been proposed to explain the existing within-tree and between years variability in FI^2,8^. Among them, the competition for carbon under high crop load conditions could explain FI inhibition. This assumption is consistent with the differential expressions of genes involved in carbon metabolism between meristems of apple trees subjected to defruited or fruited conditions^9^. Nevertheless, experimental findings have proved that the tree carbon economy is not directly involved in FI control in apple^10^. Another assumption, based on seedless varieties, considers that FI is probably affected by inhibiting signals produced by seeds of fruit, mainly gibberellins^11^ (GA). Other molecules could also be implicated to activate FI such as FLOWERING LOCUS T (FT) protein produced by leaves and considered as the florigen^8,12^.

In a previous study^10^ the impact of leaf and fruit presence on within-tree FI variability was investigated in ‘Golden Delicious’ cv. Results confirmed the existence of promoting and inhibiting signals, originating from leaves and fruit respectively. This study also showed (i) that FI was determined not only by the local conditions at the shoot scale but also by the fruit number and leaf area in the neighborhood and (ii) that the intensity of these signals strongly decreases with the distance between meristems and emitting sources. Finally and consistent with other studies^13^, the existence of signal transport in both acropetal and basipetal directions within the tree was suggested. This previous study^10^ provided a strong experimental background from which it could be possible to infer and quantify the respective effect of inhibiting and activating signals in the within tree variability in FI, as well as their combined effects and the distance at which the emitting organs can act.

Mathematical models applied to plant growth and development are promising tools to analyze the impact of hidden processes not directly accessible from experiments through model parameter fitting procedures and subsequent interpretation of parameter values^14^. In the current case of analysis of the within-tree variability in FI, functional structural plant models appear highly relevant^15^. They can combine an explicit description of plant architecture (topology and organ geometry) together with the simulation of transport of different types of molecules (water, carbon, hormones, etc.). These models rely on mathematical formalisms developed to describe and simulate plant architectural development such as multi-scale representation^16^, strings of customized plant modules in L-Systems^17^ or graphs^18^. Within-plant fluxes or molecule transports have been modeled in FSPM with a special consideration on carbon allocation^19^. It is usually assumed that assimilates are allocated depending on sink demand and distances between sources and sinks with an impact of distances modulated by empirical resistances^20–22^. More mechanistic models are based on an electric analogy for describing carbohydrate movements within the phloem^23^ or include a mechanistic modeling of coupled phloem/xylem transport^24^. Signal fluxes within plants have also been modelled, especially for the simulation of basipetal auxin transport and its consequence on bud outgrowth^25,26^ on small single stem plants, without complex branching system (*Arabidopsis thaliana*, pea). In apple tree, initial models have been proposed to simulate inhibiting and activating signal transports and their consequences on FI^27,28^. By changing manually signal quantity thresholds inducing FI, these models were promising to represent biennial bearing. However, they were not calibrated on observed data.

In this study, we built a model that simulates transports of both inhibiting and activating signals in 3D branching structures with the aim to further analyze the determinants of FI in fruit trees. This model was adapted from two previous ones, for carbon allocation^29^ and for bidirectional transport^28^. The model assumes a decrease in signal quantity with the distances from the emitting sources. FI in shoot apical meristems (SAM) was simulated based on inhibition and activation probability laws depending on both the quantity of inhibiting and activating signals. By fitting the model to a unique dataset on 3D digitized apple trees manipulated for their number of leaves and fruit^10^, we quantified the combined impact of such signals and distances at which they act on SAM and explored the underlying mechanisms that could explain within tree variability in FI.

## Material and methods

### Model overview

The model (SigFlow) was developed in python and uses libraries from the OpenAlea platform^30^. The model runs on 3D tree architectures coded in Multiscale Tree Graphs^16^ (MTG) with three scales (tree, stem segments and metamers) and augmented with organ 3D coordinates^31^. Segments are the parts of the stem between two branching points or one branching point and the axis extremity. Metamers are represented for the stem segments corresponding to the most recent shoots only and are composed of one leaf, one internode and an inflorescence if present (Supplementary Fig. S1). Fruit and leaves produce signals moving within the structure depending on the distance with an ‘attenuation’ parameter that can be tuned in order to simulate different signal distributions i.e. homogeneity within the structure or local supply. These signals reach SAM and determine their fate with probabilities depending on signal quantities. FI is simulated on the SAM located in terminal position of annual shoots. In its current version the model runs on static tree structures, consistent with our modeling aim since FI usually occurs after the end of shoot vegetative growth in adult apple trees^32^.

### Inhibiting and activating signal quantities and transport

**Figure 1:**
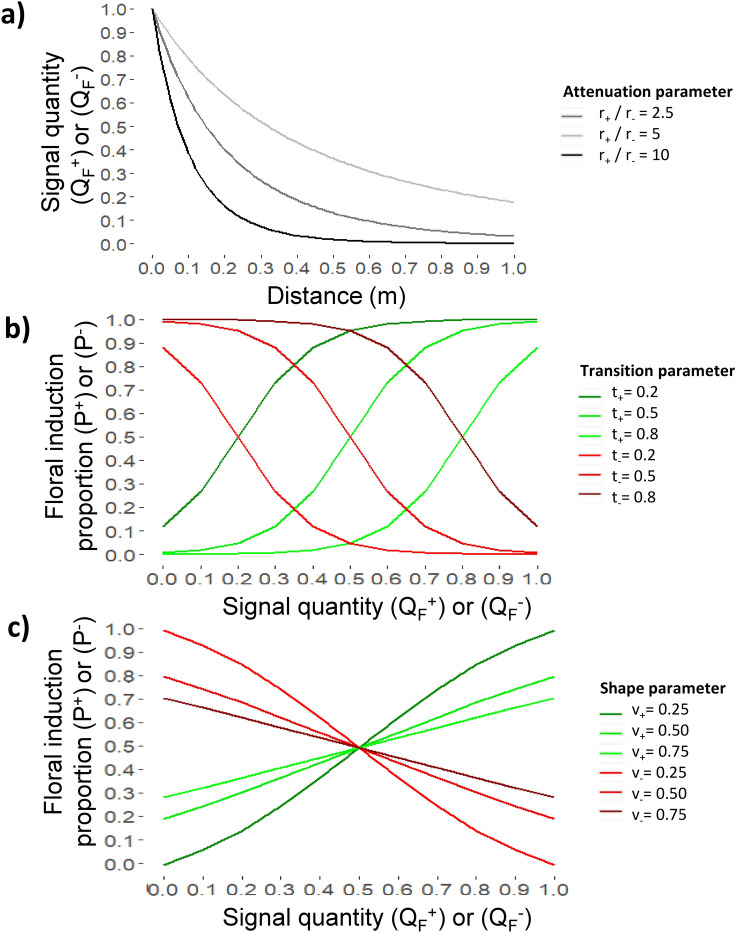
Representation of equations for signal transport and effects on floral induction. (**a**) Relationship between distance to meristems and signal quantity (activating and inhibiting) for different signal ‘attenuation’ values (*r*_*−*_*, r*_+_), (**b**) relationship between signal quantity (activating and inhibiting) and floral induction proportions for different ‘transition’ values (*t*_*−*_*, t*_+_), (**c**) relationship between signal quantity (activating and inhibiting) and floral induction proportions for different ‘shape’ values (*v*_*−*_*, v*_+_).

Equations for signal transport (inhibiting and activating) between annual shoots and SAM were adapted from previous studies dedicated to carbon allocation between sources and sinks^20,29^. These formalisms consider carbon allocation as dependent on distances between sources and sinks and on sink strength values depending on organ type and age. In our case, SAM are considered as target organs for inhibiting and activating signal with similar abilities to accumulate inhibiting or activating signal whatever the SAM. Annual shoots are considered as sources of inhibiting and activating signals originating from fruit and leaves, respectively. The quantity of signal originated from each annual shoot is computed as the sum of signals coming from each individual fruit and leaf in a given shoot. Since the equation previously proposed^29^ considered relative values of sink strength, the signal distribution from each annual shoot was rewritten to account for similar SAM abilities to accumulate signals as follows:

1 $${\text{q}}_{{{\text{ij}}}} = \frac{{{\text{Q}}_{i} \times \left( {\frac{1}{{1 + {\text{d}}_{{{\text{ij}}}} }}} \right)^{r} }}{{\mathop \sum \nolimits_{k = 1}^{n} {\text{Q}}_{{\text{i}}} \times \left( {\frac{1}{{1 + {\text{d}}_{{{\text{ik}}}} }}} \right)^{r} }}$$

where n is the number of SAM in a tree, *q*_*ij*_ is the quantity of signal exported by annual shoots (inhibiting or activating signal) *i* to SAM *j*, *Q*_*i*_ the quantity of signal produced by annual shoot *i*, *d*_*ij*_ the distance following the topological pathway between *i* and *j* and *r* an ‘attenuation’ parameter modulating the distance effect. For *r* values close to 0, the signal is equally distributed within the structure whereas it is transported at shorter distance when *r* values increases (Fig. 1a). Assuming that *r* can be different depending on the type of signal considered (inhibiting or activating signal), we defined two parameters*, r*_*−*_*, r*_+_ for the inhibiting and activating signal, respectively. We considered a normalized value equal to 1 for the inhibiting signal produced by each fruit. In order to account for a possible effect of the variations in leaf area between shoots on FI, we set the quantity of activating signal as equal to shoot leaf area. For consistency with the parameters associated with inhibiting signals, variables and parameters associated with activating signal were normalized (ranging between 0 and 1) by dividing their value by the mean shoot leaf area observed in trees.

After computations of fluxes, the quantity of signal (Supplementary Fig. S2) reaching each SAM *j* (*Q*_*F,j*_) is then computed as the total quantity of signal originated from all the sources including the shoot bearing the considered SAM:

2 $$Q_{F,j} = \mathop \sum \limits_{i = 1}^{N} \left( {{\text{q}}_{{{\text{ij}}}} } \right)$$

with N the number of annual shoots. In the following *Q*^+^_*F*_ and *Q*^*−*^_*F*_ are used for the quantity of activating and inhibiting signal, respectively.

Distances between SAMs and shoots are computed based on the organ topological position and 3D coordinates in the MTG. The distance *d*_*ij*_ between shoots and SAM (*i*, *j*) in the tree structure is computed following the topological path as the sum of the Euclidean distances between (i) the base and the barycenter of each annual shoot, (ii) plus the distances between the successive bases of the plant components and the SAMs (iii) plus the distance between the base of the annual shoot bearing the SAM and its extremity^29^.

### Computation of floral induction probability

A sigmoidal function is used to compute the probability of FI (*P*_j_) associated with the activating signal quantity produced by leaves (*P*^+^_j_) or inhibiting signal quantity produced by fruit (*P*^*−*^_j_ by the fruit) for each SAM *j*, as follows:

3 $$P^{ + }_{j} = \frac{1}{{1 { + } {\exp}\left( {\frac{{ - \left( {Q_{F,j}^{ + } - t_{ + } } \right)}}{{v_{ + } }}} \right)}}\,\,{\text{and}}\,\,P^{ - }_{j} = 1 - \frac{1}{{1 { + } {\exp}\left( {\frac{{ - \left( {Q_{F,j}^{ - } - t_{ - } } \right)}}{{v_{ - } }}} \right)}}$$

with, $t_{ + } {\text{and }}t_{ - }$ being parameters called ‘transition’ values (Fig. 1b) indicating the signal quantity (*Q*_*F*_^+^ or *Q*_*F*_^*−*^) for which SAM have 50% chance to be activated or inhibited and *v*_+_ and *v*_*−*_ parameters called ‘shape factor’ accounting from variations in the slope of the function. When *v*_+_ or *v*_*−*_ are close to 0, the probability changes rapidly from 0 to 1 when the values of *Q*_*F*_^+^ or *Q*_*F*_^*−*^ exceed or fall behind *t*_+_ or *t*_*−*_ whereas the transitions are more progressive when *v*_+_ or *v*_−_ values increase (Fig. 1c). These parameter are used to represent some uncertainty in SAM fate (floral induced or not) for a given value of inhibiting and activating signals.

Two formalisms were considered for combining the effect of fruit and leaves on FI. In the first one, FI probability (*P*_*j*_) in SAM *j* is determined by the most limiting factor only:

4 $$P_{j} = min\left[ {P^{ - }_{j} , P^{ + }_{j} } \right].$$

In the second formalism a cumulative effect of both signals determines SAM FI probability, assuming a multiplicative function:

5 $$P_{j} = P^{ - }_{j} \times P^{ + }_{j} .$$

### Model behavior assessment on simple structures

The model was applied to simple tree structures to analyze model consistency and sensitivity to parameter values. The first structure consisted of a branch composed of six shoots equidistant from each other, bearing or not leaves or fruit. Here, we aimed at testing the effect of the signal ‘attenuation’ parameter (*r*_*−*_*, r*_+_) on the quantity of signal reaching SAMs located at different distances from signal sources.

The second structure consisted of two complete branches with different spatial distributions of fruit and leaves. In the first case, fruit and leaf distributions were similar in the two branches, with half of the shoots bearing either fruit or leaves. In the second case, all the fruit or leaves were located on one branch only and the other one was completely either defruited or defoliated. Here we aimed at testing a wide range of signal ‘attenuation’ (*r*_*−*_, *r*_+_) and ‘transition’ parameter (*t*_*−*_, *t*_+_) values in order to evaluate the intertwined effects created by the model, between tree architecture, signal transport and SAM sensitivity to the quantity of signals.

Lastly, we analyzed model formalisms (“limiting factor”, “multiplicative formalism”; Eqs. 4 and 5) used to integrate the combined effects of inhibiting and activating signals on SAM FI. We used a structure composed of four contrasted branches, i.e. a leafy and fruiting branch, a foliated branch without fruit, a fruiting branch without leaf and a defoliated and non-fruiting branch. The two formalisms were used with a wide range of ‘transition’ values (*t*_*−*_*, t*_+_) to modify SAM sensitivity to both signals.

### Description of the experimental dataset

Data used for building the tree 3D representations and for calibration and validation purpose were taken from experiments carried out in 2017 on 10-year-old apple trees (cv. ‘Golden Delicious’) orchard, located at the SudExpé experimental station in Marsillargues, in the south of France (43° 66′ N 4° 18′ E). In this experiment described in Belhassine et al.^10^, leaves and/or fruit were removed in different parts of the tree (Supplementary Fig. S2). On trees set in ON (high fruit load) or OFF (crop load close to 0) conditions, fruit or leaves were removed on half of the shoots and half of the branches of trees trained as “solaxe” (one main vertical trunk) and on one side of trees with a Y-Shape (two main trunks). An additional set of trees not subjected to fruit or leaf removal but displaying a natural variability in crop load were observed during three years (2015–2016–2017).

SAM FI proportion in trees was estimated at full bloom in the spring following treatment onset, as the ratio of the total number of reproductive buds to the total number of growing buds in the different parts of the trees (leafy, non-leafy or fruiting, non-fruiting). Tree crop load was estimated as the fruit number at harvest divided by the trunk cross sectional area (TCSA, cm^2^) estimated in autumn after fruit harvest^33^.

### Input architectures

Architecture description was performed on one “solaxe” tree and one Y-shape tree displaying TCSA values (20.4 and 24.7 cm^2^, for solaxe and Y-Shape trees, respectively) close to the mean values observed in the orchard. 3D coordinates were acquired using an electromagnetic 3D digitizer (3Space Fastrak; Polhemus Inc., Colchester, VT, USA) at the trunk base, branching points and top and bases coordinates of each annual shoot (Supplementary Fig. S1). The 3D structures, including tree entities organized in three topological scales and their coordinates, were saved in MTG format. These structures were used to reconstruct leaf location and area along annual shoots and to reproduce in silico experimental treatments of leaf and fruit removal. Leaf area distribution along annual shoots was reconstructed based on allometric relationships as previously proposed^34^ (Supplementary Methods S1). To build allometric relationships, data were collected on both 60 short (< 5 cm) and long shoots (> 5 cm) and individual leaf areas were estimated with a leaf area meter (LI 3100 Area Meter, LI-COR, Lincoln, NE, USA) every three leaves along the shoots. Mature leaves were sampled after fruit harvest to build these allometric relationships. On ON trees, one fruit was added in the MTG at the base of each annual shoot (consistent with thinning practices in the field).

### Estimation of parameter values and model assessment

Model outputs extend the MTG with new attributes for each SAM *j* corresponding to the quantities of activating and inhibiting signals reaching it after transport (Q^+^_*F,j*_ and Q^−^_*F,j*_), its probability of FI associated with received activating (*P*^+^_*j*_) or inhibiting signals (*P*^*−*^_*j*_) and its final FI probability combining both signals (*P*_*j*_). Parameters associated with either activating or inhibiting signal effects on FI were estimated separately on leaf and fruit removal treatments, respectively. Activating signal parameters (*r*_+_, *t*_+_, *v*_+_) were estimated on non-fruiting structures subjected to leaf removal at different scales (shoot, branch, and half-tree) or not (control OFF trees). Inhibiting signal parameters (*r*_*−*_, *t*_*−*_, *v*_*−*_) were estimated on leafy and fruiting tree structures subjected to fruit removal at different scales (shoot, branch and half-tree) or not (control ON trees) and on leafy non-fruiting trees (control OFF trees). Simulated FI proportions (i.e. proportion of meristems that were floral induced) were compared to the observed FI ones in the different parts of the trees (leafy or non-leafy, fruiting or non-fruiting). Simulated proportions were computed as the average FI probability of each bud in the different parts of the trees. The parameter values of the simulation displaying the lowest error between simulated and observed FI for all conditions (tree and local treatments) were selected as the best solution in the calibration procedure. Two steps in the calibration procedure were done. A first step consisted in exploring a wide range of values by varying *r*_+_ and *r*_*−*_ from 0 to 15 (step = 0.1), *t*_+_*, t*_*−*_ from 0 to 1 (step = 0.1) and *v*_+_ and *v*_*−*_ between 0.01, 0.1, 0.3 and 0.6 for activating and inhibiting signals respectively (6,644 simulations in total). In a second step, the range of values close to the best solutions was narrowed down to refine estimations (1,155 simulations).

Model validations were performed in two steps. First, the two sets of parameter values for activating and inhibiting signals obtained from calibration were used to simulate FI probability in fruiting trees subjected to leaf removal. The two functions which represent the combined effect of both signals on SAM FI, i.e. with a “limiting factor” or a multiplicative formalism, were compared through simulated SAM FI proportions. Second, validations were performed on the digitized “solaxe” tree on which contrasted crop loads were obtained by in-silico fruit removal. FI proportions simulated for these different in silico crop loads were compared to the relationship between FI and crop load obtained from the additional trees displaying contrasting crop load in the experiment.

Model calibration and validation quality was evaluated using root mean square error (RMSE), bias (absolute sum of differences divided by replicate number) and R^2^ between observed and simulated values.

## Results

### Model behavior and sensitivity to model parameters

**Figure 2:**
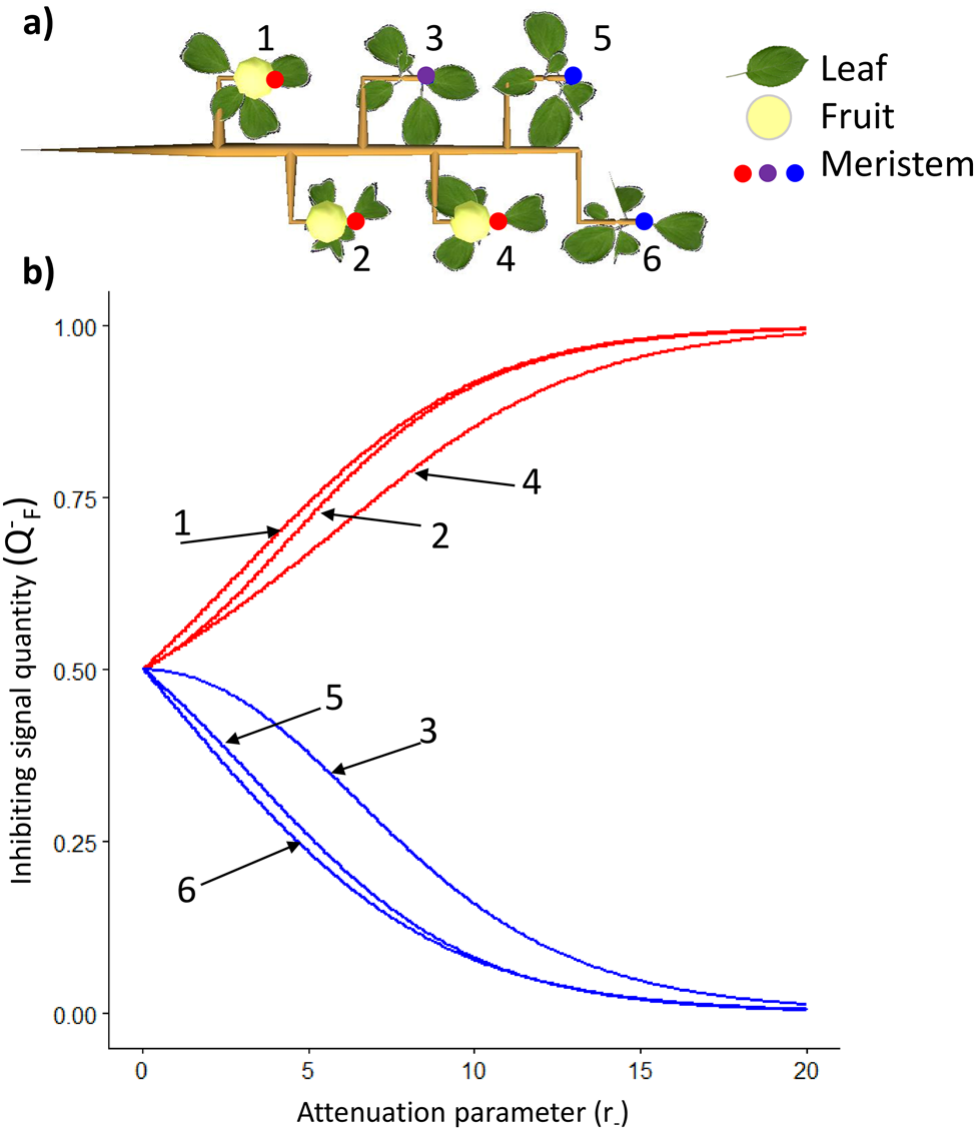
Inhibiting signal concentration (*Q*^*−*^_*F*_) in six meristems for different values of the signal ‘attenuation’ parameter (*r*_*−*_) (**b**). Simulations were performed on a simple structure composed of six shoots and three fruit, each shoot being located at 15 cm from each other (**a**). 1, 2 and 4 are fruiting shoots and 3, 5 and 6 are non-fruiting shoots.

The ‘attenuation’ parameter of the inhibiting signal (*r*_−_) was varied on a simple structure composed of six shoots located at an equal distance from each other (15 cm) and with three sources (fruit) (Fig. 2a). When no signal ‘attenuation’ (*r*_−_ = 0) was considered, inhibiting signal was equally transported to the six SAMs (Fig. 2b): each one had an inhibiting signal value equal to 0.5 which represented the number of fruits divided by the SAM number. When *r*_−_ values were increased, the quantity of inhibiting signal reaching each meristem (*Q*^−^_*F*_) increased and decreased in SAM of fruiting and non-fruiting shoots, respectively. For the highest values of *r*_−_ (roughly over 15) *Q*^−^_*F*_ was equal to 0 in the SAM of non-fruiting shoot and 1 in the ones of fruiting shoots. When considering medium values of *r*_−_, differences between fruiting and non-fruiting shoot resulted from the distance of each shoot to the other ones. Among non-fruiting shoots, the ranking of *Q*^−^_*F*_ in SAM depended on its distance to all fruit, with SAM of shoot 3 (sum of distances to the fruit = 45 cm) displaying the highest quantity, SAM 6 (90 cm) the lowest and SAM 5 (75 cm) a medium value. Similarly, SAM 1 and 2 of fruiting shoots displayed higher *Q*^−^_*F*_ than SAM 4 because they were located close to each other and could exchange inhibiting signal. In another simulation set, the sources of activating (leaves) signals were varied on the same simple structure considering leafy and non-leafy shoots to analyze the effect of attenuation parameter associated with activating signal (*r*_+_) on activating signal quantity in SAM (*Q*^+^_*F*_, Supplementary Fig. S3). Similar results for the signal quantities variations in SAM were obtained since model assumptions are symmetric for both inhibiting and activating signals.

**Figure 3:**
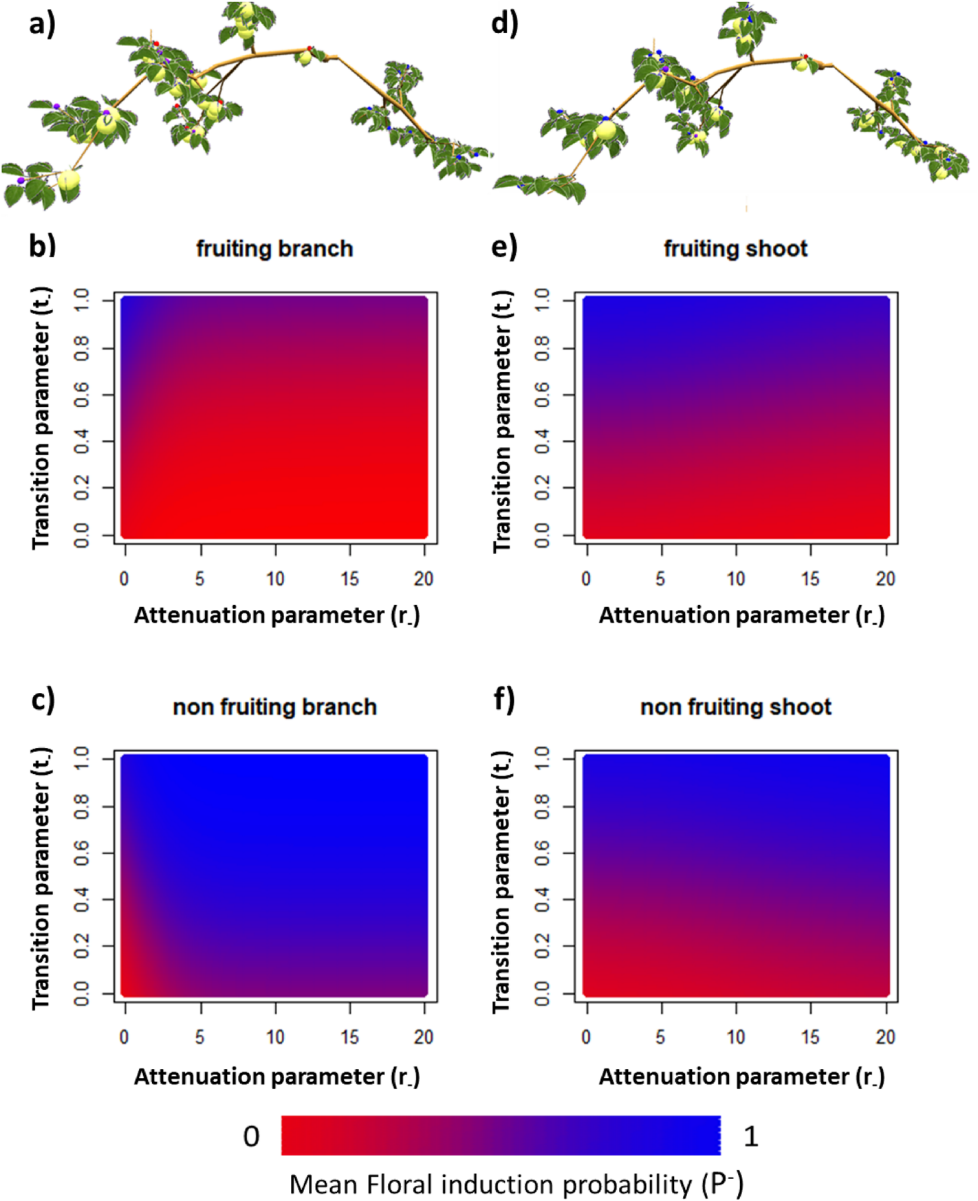
Mean floral induction probability (*P*^*−*^) in shoot apical meristems depending on the quantity of inhibiting signal produced by fruit for different values of the signal ‘attenuation’ (*r*_*−*_) (200 values) and ‘transition’ parameters (*t*_*−*_) (100 values). Simulations were performed on two hypothetical structures composed of two branches with one fruiting and one non-fruiting branch (**a**) and two branches with homogeneous fruit removal on half of the shoots (**d**). (**b**) and (**c**) represent the mean floral induction proportion in fruiting and non-fruiting branch, respectively for the structure represented in (**a**). (**e**) and (**f**) represent the mean floral induction proportion in fruiting and non-fruiting shoots, respectively for the structure represented in (**d**). Simulations were performed assuming a shape parameter value (v_−_) equal to 0.25.

The impact of the signal ‘attenuation’ (*r*_*−*_) and ‘transition’ parameters (*t*_*−*_) on SAM floral induction probability depending on inhibiting signal (*P*^*−*^) was assessed on simple structures composed of two branches with contrasted location of fruit (random fruit removal on both branches, Fig. 3d; one fruiting and one non-fruiting branch, Fig. 3a). On the two structures and except for r_−_ = 0 (homogeneous distribution of signal between each SAM), *P*^−^ was higher in SAM located on non-fruiting branches or shoots than in SAM located in fruiting branches or shoots (Fig. 3b,c). The contrast in *P*^*−*^ between fruiting branches/shoots and non-fruiting ones increased when *r*_*−*_ values increased due to a transport of the inhibiting signal at shorter distances (Fig. 3b,c) Moreover, the contrast in *P*^*−*^ between fruiting and non-fruiting parts was lower when fruit were removed randomly on both branches (Fig. 3e,f) than when removal was performed at the branch scale (Fig. 3b,c). This results from the lower distances between fruiting and non-fruiting shoots when fruit removal was performed on half of the shoots than between fruiting and non-fruiting branches when fruit removal was performed at the branch scale. The effect of *t*_*−*_ parameter (indicating the signal quantity threshold for which *P*^*−*^ was equal to 50%), was consistent with its expected impact as *P*^*−*^ increases when *t*_*-*_ increases. As expected from model equations, *P*^*−*^ was equal to 1 in all conditions when *t*_*−*_ was equal to 1. Model behavior was similar when considering the impact of leaf removal at different scales (shoot or branch, Supplementary Fig. S4) on floral induction probability associated with the activating signal (*P*^+^) on the same two-branches structures subjected to leaf removal since model hypotheses for inhibiting and activating signal are similar.

**Figure 4:**
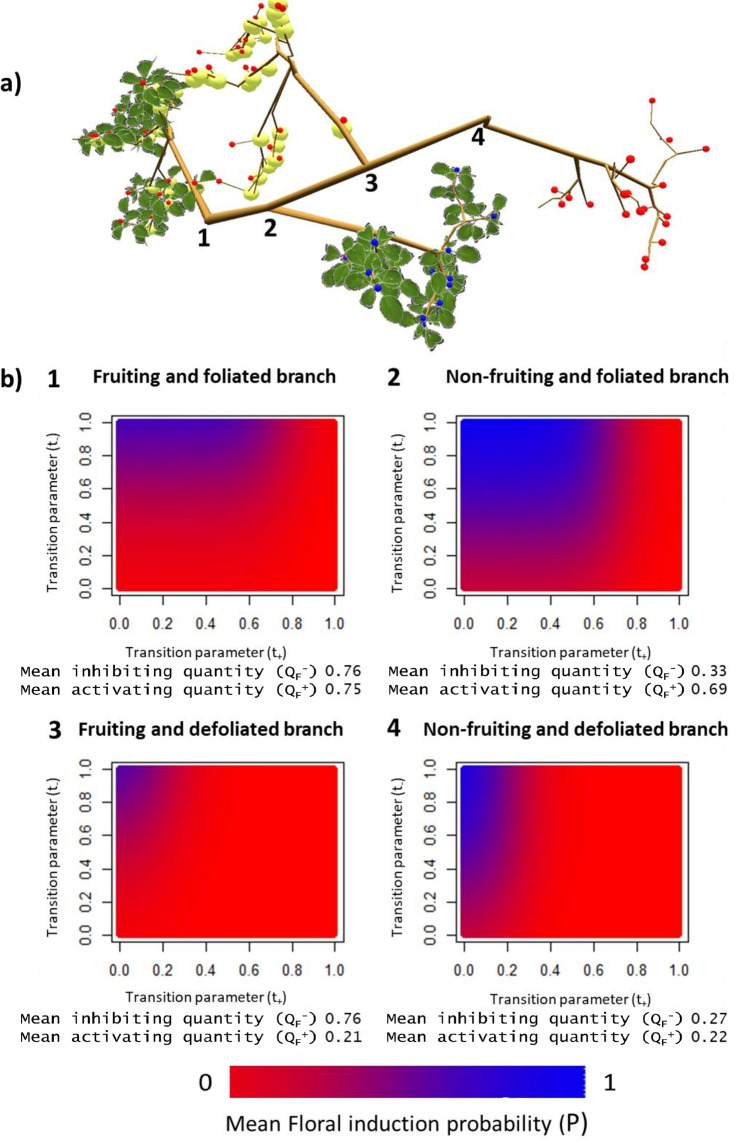
Mean floral induction probability (*P*) in shoot apical meristem for different ‘transition’ parameter values for the activating (*t*_+_) and inhibiting (*t*_*−*_) signals (100 × 100 values). Floral induction proportions were computed on a hypothetical structure composed of one fruiting and leafy branch (1), one non-fruiting and leafy branch (2), one fruiting and non-leafy branch (3) and one non-fruiting and non-leafy branch (4) (**a**). (**b**) represents the mean floral induction proportion in the different branches. Mean inhibiting and mean activating below the heatmaps represent the mean of the inhibiting and activating signals quantities for all meristems in each branch. Simulations were performed assuming a multiplicative effect of the inhibiting and activating signal on floral induction. In these simulations ‘shape’ parameters (*v*_*−*_, *v*_+_) equal to 0.25 and ‘attenuation’ parameters (*r*_+_, *r*_*−*_) equal to 2.5 were used.

Simulations were then performed to analyze model behavior when effects both of leaves and fruit are taken into account. In these simulations, resulting FI probabilities (*P*) were calculated by either the function assuming a limiting-factor (Supplementary Fig. S5) or the multiplicative (Fig. 4) formalism on simple structures composed of four branches bearing leaves and fruit or not. In these simulations, signal (inhibiting and activating) transport could occur between branches to simulate some inhibiting and activating signal quantities in non-fruiting and non-leafy branches, respectively. When medium values for ‘transition’ parameters (*t*_*−*_ = *t*_+_ = 0.5) were considered, *P* was, as expected, the highest in non-fruiting and leafy branches, i.e. in presence of activating signal and in absence of inhibiting one, whatever the chosen function (multiplicative, Fig. 4 or limiting factor formalism, Supplementary Fig. S5). For the three other branch configurations and whatever the formalism, *P* were low due to either high inhibiting signal quantity in fruiting branches (Fig. 4b, 1,3; Supplementary Fig. S5b, 1,3) or low activating signal quantity in non-leafy branches (Fig. 4b, 3,4; Supplementary Fig. S5b, 1,3). When varying the transition parameter values, lower *P* were simulated by the multiplicative formalism than by the limiting factor one. This was mainly observed for fruiting and foliated branches (Fig. 4b, 1 and Supplementary Fig. S5b, 1) and non-fruiting and defoliated branches (Fig. 4b, 4 and Supplementary Fig. S5b, 4) when t_−_ < 0.7 and t_+_ > 0.3, respectively. In those cases, an additional effect on *P* of the less-limiting factor (activating signal in foliated branches, or inhibiting signal in non-fruiting branches) was simulated by the multiplicative formalism. This effect was due to fruit presence in neighboring branches in the non-fruiting branches or leaf absence in the neighborhood for the leafy ones.

### Model calibrations and associated parameters values

**Figure 5:**
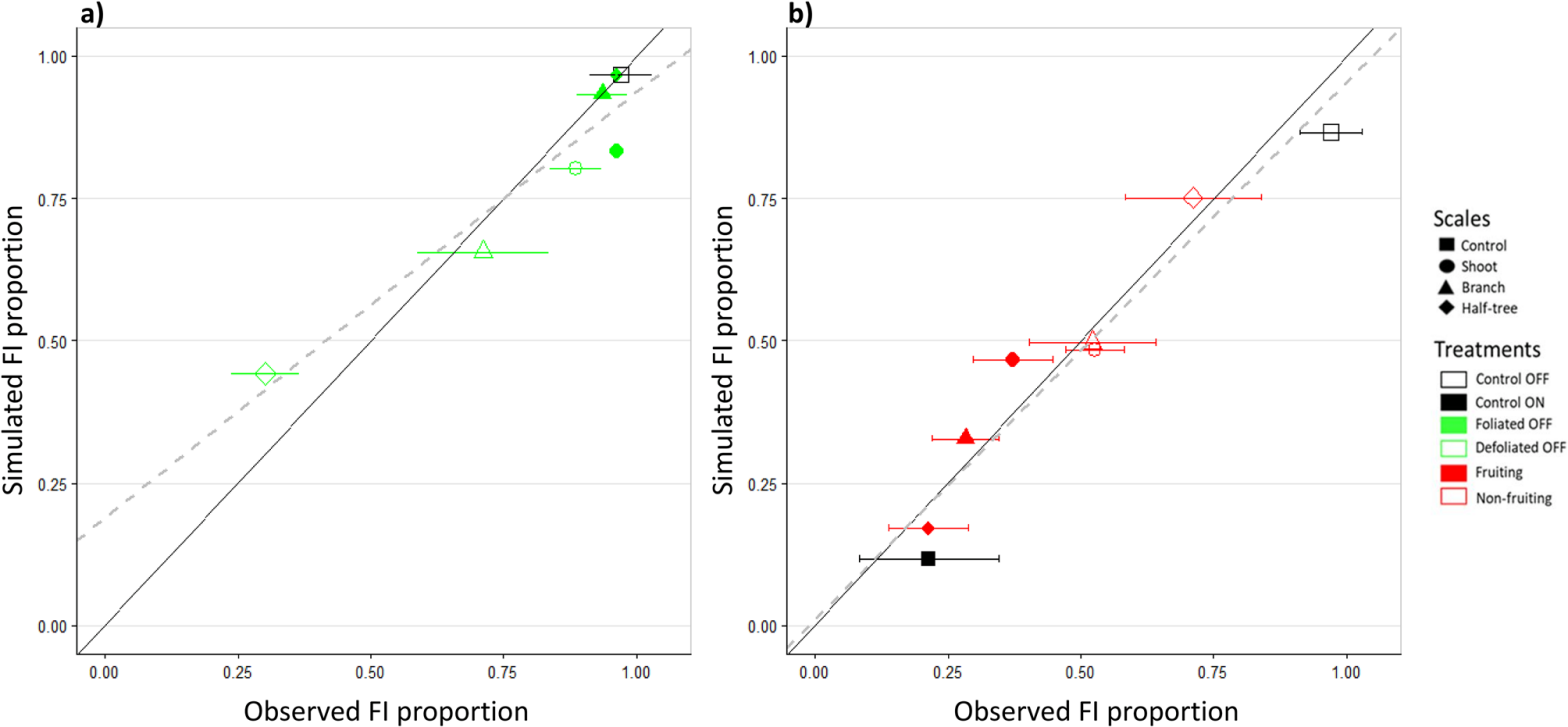
Observed and simulated floral induction proportions for the treatments used for calibration. Parameters associated with either activating or inhibiting signal effects on floral induction were estimated separately on leaf (**a**) and fruit removal (**b**) treatments. Treatments were performed by removing fruit or leaves on half of the shoot (shoot scale), half of the branches (branch scale) or on one side of trees with a Y-Shape (half-tree scale). Bars represent the observed standard deviation in floral induction proportion (3 tree replicates). Continuous lines represent the 1:1 lines and the dashed ones are the linear fits between observed and simulated proportions (R^2^ = 0.90 and 0.93 for (**a**) and (**b**), respectively).

Model calibrations performed separately on trees with leaf or fruit removal were highly relevant when confronting observed and simulated FI proportions in the different parts of the trees subjected to local fruit and leaf removal (Fig. 5). R^2^ and RMSE were equal to 0.90 and 0.116 and 0.93 and 0.059, for the calibration performed on trees with leaf or fruit removal, respectively.

This calibration procedure resulted in a set of estimated parameters related to either activating or inhibiting signal. The estimated ‘transition’ parameter value (*t*, Table 1) was lower for the activating (0.09) than for the inhibiting signal (0.47), revealing that low quantities of activating signal were enough to trigger FI. The estimated signal ‘attenuation’ (*r*) value was higher for the activating signal (5.0) than for the inhibiting one (2.7). These values correspond to roughly less than 10% of the emitted signal reaching a SAM when located at more than 0.5 m or 1.2 m from sources of activating and inhibiting signals (leaves and fruit), respectively (Supplementary Fig. S6). Estimated ‘shape’ parameter values were similar for both activating and inhibiting signals (0.25) and account for a noticeable uncertainty in SAM fate for a given quantity of signal. Indeed, although the ‘transition’ value for the inhibiting signal (0.47) represents the quantity of signal needed to reach a FI probability of 0.5, this probability was equal to 0.27 when the quantity of inhibiting signal was high (0.75) and still remained non null (0.11) for an quantity of signal equal to 1 (Supplementary Fig. S6).

**Table 1:** Values of ‘attenuation’ (r_+_ , r_−_), ‘transition’ (t_+_ , t_−_) and ‘shape’ (v_+_ , v_−_) parameters estimated for activating and inhibiting signals respectively and RMSE, R^2^ and bias between observed and simulated FI proportion.

Calibration dataset											
Activating signal						Inhibiting signal					
r_+_	t_+_	v_+_	RMSE	R^2^	Bias	r_−_	t_−_	v_−_	RMSE	R^2^	Bias
5.0	0.09	0.25	0.116	0.90	0.072	2.7	0.47	0.25	0.059	0.93	0.001

**Figure 6:**
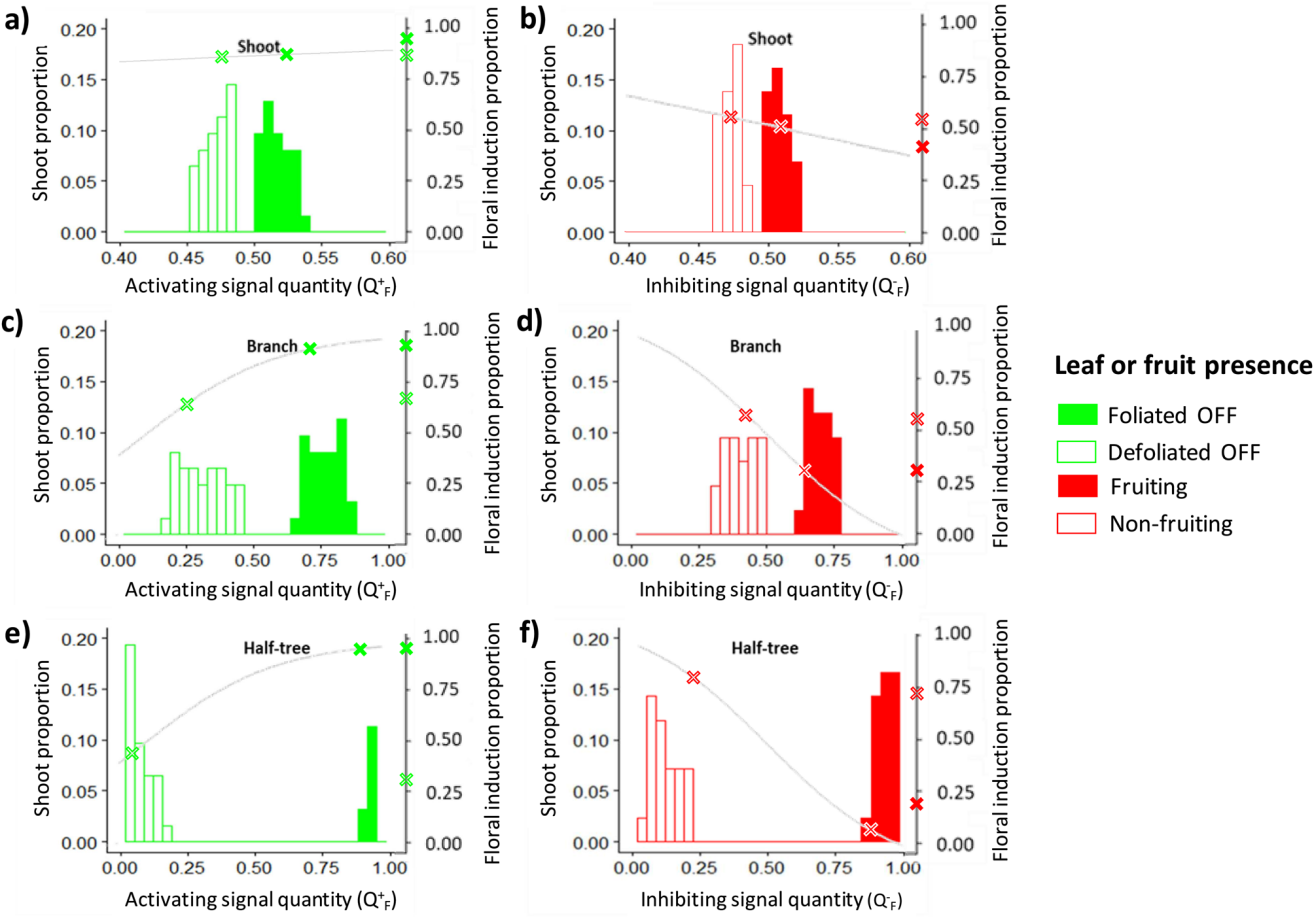
Distribution of simulated signal quantity in shoot apical meristem and resulting floral induction proportions depending on activating (**a**,**c**,**e**) and inhibiting (**b**,**d**,**f**) quantities in the different experimental conditions. Treatments were performed by removing fruit or leaves on half of the shoot (**a**,**b**), half of the branches (**c**,**d**) or on one side of trees with a Y-Shape (**e**,**f**). Shoot proportions depending on signal quantity are represented by bars and the resulting FI probability with a continuous grey curve. Crosses on Y axes and grey curves represent the observed and simulated FI proportions, respectively. Blank and colored crosses are used for the defoliated/defruited tree parts and foliated/fructified parts, respectively.

Distributions of simulated FI probability depending on the quantities of inhibiting and activating signals in the different SAMs of trees subjected to either leaf or fruit removal provided additional information about the underlying signal and distance effects FI probability in the different tree parts (Fig. 6). Inhibiting and activating signal quantity were slightly lower in leafy compared to non-leafy parts and in non-fruiting parts compared to fruiting ones when removals were performed at the shoot scale (Fig. 6a,b). These small differences were consistent with the observed low differences in FI proportion between leaf/non-leafy or fruiting/non-fruiting shoots and resulted from the short distances between neighboring shoots within tree structures. Among shoots of a given type (leafy, non-leafy, fruiting, non-fruiting), variations in the quantity of inhibiting and activating signals were quite low when treatments were performed at the shoot scale. This likely resulted from (i) the low variation in the observed individual shoot leaf areas (e.g. more than 75% of the shoots had leaf area between 80 and 120 cm^2^ in the “solaxe” tree, Supplementary Fig. S7) and (ii) because of the lack of variation in the fruit number per shoot (all shoots were considered as bearing one fruit in ON trees).

Differences in signal quantity (*Q*_*F*_) between the different tree parts were stronger when organ removals were considered at coarser scales (branch or half-tree) with a greater impact of leaf/fruit removals on half-trees, consistent with the increasing distances to the remaining fruit or leaves (Fig. 6c–f). These differences were of greater extent for leaf removal than for fruit removal due to the higher estimated signal ‘attenuation’ parameter value for the activating signal (*r*_+_) than for inhibiting one (*r*_−_) (Table 1). Nevertheless, and consistently with observed data, the simulated impact of leaf removal on FI probability was lower than that of fruit due to lower estimated ‘transition’ parameter (*t*) values for the activating signal. Finally, quite large variability in signal quantities was obtained in the different SAMs of non-leafy (*Q*^+^_*F*_) and non-fruiting parts (*Q*^*−*^_*F*_) when treatments were performed at the branch or half tree scale. This variability probably resulted from the spatial distribution of SAM within the tree, with SAM at branch bases closer to the sources of signals coming from other branches than ones located at branch extremities.

### Signal combined effect and model validation

**Figure 7:**
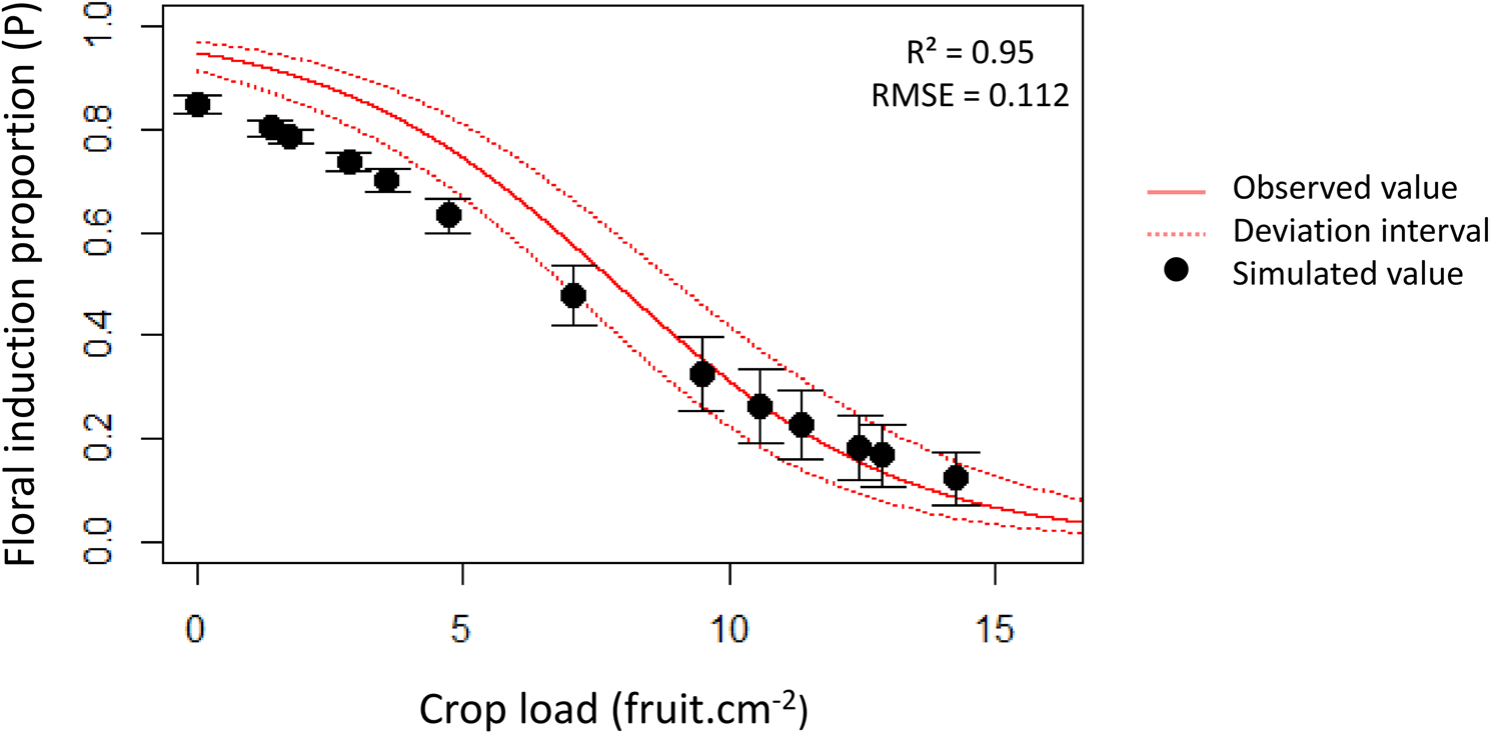
Simulation of floral induction proportions depending on crop load. Black points represent the simulated floral induction proportion for different values of crop loads using the set of parameters estimated after the calibration procedure. The continuous line represents the result of the exponential adjustment performed on experimental data (y = $\exp ( - 0.3721 \times {\text{x}} + 2.9273)/\left( {1 + \exp \left( { - 0.3721 \times {\text{x}} + 2.9273} \right)} \right)$. The relationship was built on “additional” control trees in 2015, 2016 and 2017. The dotted red lines represent the deviation interval of the fitted values. Bars represent the within tree standard deviation of FI in the simulations.

Model validation was performed on ON trees subjected to leaf removal at different scales (shoot, branch and half-tree) by using the set of parameters previously estimated and with the two formalisms proposed to account for the combined effect of activating and inhibiting signals (limiting factor or multiplicative formalism, Table 2). Bias values showed that both formalisms underestimated the effect of signal combination on proportion of meristems that were floral induced. However, the differences among treatments were better simulated (R^2^ = 0.47) with the multiplicative formalism. Nevertheless, the range of variation in the observed FI proportions was low, with FI proportion close to 0 in all the treatments considered. We thus performed another set of in-silico experiments to complement the validation step. Trees with contrasted crop loads (ratio of harvested fruit number to trunk cross sectional area) were represented by removing varying proportion of fruit on ON trees. FI proportion (simulated with the multiplicative formalism) were in overall adequate agreement with the estimated relationship with tree crop load, built from field observations (R^2^ = 0.95, RMSE = 0.112) (Fig. 7). Model outputs were in agreement with observations for crop load values higher than 5 fruit cm^−2^ and tended to underestimate FI proportions for lower crop load values. Nevertheless, differences between observed and simulated FI proportions remained lower than 10%.

**Table 2:** RMSE, R^2^ and bias between observed and simulated FI proportion for the validation dataset and for the limiting factor or multiplicative formalism.

Validation dataset						
Range of variation (FI proportion observed value)	Limiting factor			Multiplicative formalism		
	RMSE	R^2^	Bias	RMSE	R^2^	Bias
0.03–0.15	0.089	0.004	0.036	0.055	0.47	0.063

Validations were performed using the calibrated sets of parameter values for activating and inhibiting signals. Simulations were performed on fruiting trees subjected to leaf removal at different scales (shoot, branch and half-tree).

## Discussion

### A model-based approach for quantifying the respective roles of leaves and fruit on FI

This study proposes a new model (SigFlow) that simulates for the first time FI variability in a fruit tree as the result of the combined effects of its architecture, inhibiting and activating signals and SAM sensitivity to these signals. The model proved its ability to represent experimental observations in which the within-tree distribution of inhibiting and activating sources were artificially modified^10^. It was also able to rebuild the relationship between tree crop load and the proportion of flowering SAMs in the next year^35^. The model assumes that signal attenuation is related to distances from emitting sources and that FI could be determined using probability functions depending on the combination of both signal quantities. It uses a generic formalism adapted for representing all types of plant architectures (MTG^16^). The proposed model could thus be applied to other fruit tree species in which FI varies within the tree and between years (e.g. peach, olive, plum and walnut trees). This study focused on FI proportion in SAM located in terminal position on annual shoots and did not account for meristem fate in axillary position along medium and long shoots. This simplification is relevant for adult apple trees displaying short shoots mainly^36^. In the case of younger trees or other fruit trees bearing buds in axillary positions (e.g. *prunus* sp.), our model would also be relevant for computing axillary flowering probability but it should be complemented with other sub-models to account for the position of axillary meristems in the flowering zones along the parent shoot^37^. Generating potential floral zone in the tree structure could be achieved using statistical models, as done previously^38^, or more mechanistically by simulating shoot growth dynamics within the growing season^39^. Indeed, FI occurrence was observed to be associated with a decrease in plastochron and shoot growth cessation^40,41^.

Previous models for simulating signal transport in plants were developed to represent basipetal auxin polar transport at short distances within monopodial stem^25,26^. Our approach relied on a more general formalism previously proposed for source-sink models to represent fruit growth variability^21,22,29^. In our model, no preferential direction in signal fluxes was assumed as previous studies showed that fruit and leaf influence occurs in both acropetal and basipetal directions^13^. In this tree structure, the quantity of inhibiting signal produced by each fruit was assumed to be similar, since it is associated with a constant seed number per fruit (source of GA) for a given genotype. The quantity of activating signal was assumed to be associated with shoot leaf area values based on previous results showing the close relationship between shoot vigor or length and FI^6^.

### Parameter estimation leads to new assumptions on the physiological process involved in the within-tree variability in floral induction

A heuristic approach was carried out by adjusting parameter values (‘attenuation’, ‘transition’ and ‘shape’ parameters) to a set of experimental data. The estimated parameter values allowed us to propose new hypotheses explaining the within tree variability in FI in apple trees.

The signal ‘attenuation’ parameters represent the distance effect and the decrease in the influence of fruit and leaves. It could be associated with the rate of hormone or protein accumulation or degradation during their transport from the emitting sources to SAM^42^. The estimated values of ‘attenuation’ parameters were relatively higher for the activating signal than for the inhibiting one. This result is consistent with the relative short distance of the action of the florigen, i.e. FT protein, in fruit trees^43,44^. Molecules other than FT could have an activating effect on FI. Among them sugar signaling molecules such as trehalose-6-phosphate were observed to affect flowering time in Arabidopsis thaliana by inducing FT production^45^. The present study also provides quantitative support to the assumption of the relatively long-distance transport of an inhibiting signal originating from fruit that could correspond to a GA mobile form, especially GA12 that was observed to be transported in small plants^46,47^. However, molecules other than GA could also be involved such as auxin that can act as a second messenger and can be transported over long distances^8^.

The ‘transition’ parameter represents the sensitivity of SAMs to the quantity of signals they receive. The estimated values suggest that a low quantity of activating signal corresponding to 33 cm^2^ of shoot leaf area only (*t*_+_ = 0.12 when normalized values were used) is enough to activate FI. Such an area is low compared to the range of shoot leaf areas observed in apple tree^48^. This means that in most agronomic cases and for trees not subjected to any drastic defoliation, the quantity of signal produced by leaves does not limit FI. This result suggests to reinterpreting the observed positive correlation between shoot leaf area and FI^5,6^. Increasing leaf area does not likely activate by itself FI. The relationship between shoot leaf area and FI could thus result from a longer distance between fruit and SAM that may in turn reduce inhibiting signal quantity reaching SAM. In apple trees, large genotypic variability in shoot length exists^49^ that has been associated with a higher tendency to return bloom each year in cultivars bearing fruit on long shoots than those bearing only spurs (short shoots). Such relationships seem to disappear when exploring the variability within a segregating population^50^. In that context, it is likely that each genotype may have different physiological regulations, for instance different quantity of inhibiting signal produced in relation for instance with the observed genotypic variability in seed number per fruit^51^. The modeling approach proposed, integrating both architectural and functional traits (quantity of signal, signal transport, meristem sensitivity), could be a promising way to explore the determinants of the genotypic variability in FI.

Model outputs support a combined effect of activating and inhibiting signals on FI since the multiplicative formalism, even though not stringent enough, was better at simulating FI in fruiting and non-leafy parts of the trees than the formalism based on a “limiting factor”. This can be interpreted as the likely implication of both signals in a common pathway responsible for FI. This assumption is consistent with previous studies showing an impact of GA on the floral pathway integrator SOC whose activity is also regulated by FT^52,53^.

Finally, the high values of the shape parameter representing the level of uncertainty in the SAM FI for a given quantity of signals reveal some limitations of our modeling approach to represent FI within an apple tree structure. It is likely that the model hypotheses, assuming a role of leaves and fruit only, are not sufficient to represent all the complex processes involved in within tree variability in FI. Indeed several pathways are known to be involved, including climatic conditions, in particular temperature^54^, which can be modified by the microclimate conditions within the canopy. Moreover, in our model we did not consider any preferential direction in signal fluxes (acropetal or basipetal) which could locally affect FI proportion. Although it is known that GA^46^ and FT^55^ can move in the vascular xylem–phloem system, no results exist on a possible preferential transport for the signaling molecules (with the transpiration and water flux or with the phloem mass flow).

## Conclusion

In this study, we developed a new generic model (SigFlow) for simulating the transport of inhibiting and activating signals within tree structures that was calibrated using a unique set of experimental data in apple trees. Although the nature of the signal remains to be elucidated, the estimation of model parameter values and the comparisons of two signal combining functions allowed us to propose new assumptions regarding the respective influences of inhibiting and activating signals and the distance effects in the determination of FI. Model outputs support the hypothesis that inhibiting and activating signals interact to determine FI, with the SAM being more sensitive to inhibiting signal than activating one, and that fruit signals act at longer distances than leaves. Moreover, leaf area in actual agronomic conditions is likely non-limiting for FI. This model thus opens new perspectives to understand further the physiological and architectural determinants of FI in trees.

## Supplementary information

Supplementary Information.

## Data Availability

The dataset is available from the corresponding author upon request. Model implementation and input architectures are available from SigFlow open-source repository through the OpenAlea platform (https://github.com/openalea/sigflow).

## References

[1] Monselise SP, Goldschmidt EE (1982). Alternate bearing in fruit trees. Hortic. Rev..

[2] Wilkie JD, Sedgley M, Olesen T (2008). Regulation of floral initiation in horticultural trees. J. Exp. Bot..

[3] Samach A, Smith HM (2013). Constraints to obtaining consistent annual yields in perennials. II: Environment and fruit load affect induction of flowering. Plant Sci..

[4] Costes E, Lauri P-E, Regnard J-L (2006). Analyzing fruit tree architecture: Implications for tree management and fruit production. Hortic. Rev..

[5] Neilsen JC, Dennis FG (2000). Effects of seed number, fruit removal, bourse shoot lenght and crop density on flowering in ‘spencer seedless’ apple. Acta Hortic..

[6] Lauri P-E, Trottier C (2004). Patterns of size and fate relationships of contiguous organs in the apple (*Malus domestica*) crown. New Phytol..

[7] Palmer JW, Cai Y-L, Edjamo Y (1991). Effect of part-tree flower thinning on fruiting, vegetative growth and leaf photosynthesis in ‘Cox’s Orange Pippin’ apple. J. Hortic. Sci..

[8] Hanke M-V, Flachowsky H, Peil A, Hättasch C (2007). No flower no fruit—genetic potentials to trigger flowering in fruit trees. Genes Genomes Genom..

[9] Guitton B (2016). Analysis of transcripts differentially expressed between fruited and deflowered ‘Gala’ adult trees: A contribution to biennial bearing understanding in apple. BMC Plant Biol..

[10] Belhassine F (2019). Impact of within-tree organ distances on floral induction and fruit growth in apple tree: Implication of carbohydrate and gibberellin organ contents. Front. Plant Sci..

[11] Dennis FG, Neilsen JC (1999). Physiological factors affecting biennial bearing in tree fruit: the role of seeds in apple. HortTechnology.

[12] Corbesier L, Coupland G (2006). The quest for florigen: A review of recent progress. J. Exp. Bot..

[13] Haberman A (2016). Different flowering response to various fruit loads in apple cultivars correlates with degree of transcript reaccumulation of a TFL1-encoding gene. Plant J..

[14] Cournède P-H (2011). Some parameter estimation issues in functional–structural plant modelling. Math. Model. Nat. Phenom..

[15] Vos J (2009). Functional–structural plant modelling: A new versatile tool in crop science. J. Exp. Bot..

[16] Godin C, Caraglio Y (1998). A multiscale model of plant topological structures. J. Theor. Biol..

[17] Lindenmayer A (1968). Mathematical models for cellular interactions in development. II Simple and branching filaments with two-sided inputs. J. Theor. Biol..

[18] Hemmerling R, Kniemeyer O, Lanwert D, Kurth W, Buck-Sorlin GH (2008). The rule-based language XL and the modelling environment GroIMP illustrated with simulated tree competition. Funct. Plant Biol..

[19] Lacointe A (2000). Carbon allocation among tree organs: A review of basic processes and representation in functional–structural tree models. Ann. For. Sci..

[20] Balandier P (2000). SIMWAL: A structural–functional model simulating single walnut tree growth in response to climate and pruning. Ann. For. Sci..

[21] Lescourret F, Moitrier N, Valsesia P, Génard M (2011). QualiTree, a virtual fruit tree to study the management of fruit quality. I. Model development. Trees Struct. Funct..

[22] Pallas B (2016). Simulation of carbon allocation and organ growth variability in apple tree by connecting architectural and source-sink models. Ann. Bot..

[23] Allen MT, Prusinkiewicz P, DeJong TM (2005). Using L-systems for modeling source-sink interactions, architecture and physiology of growing trees: The L-PEACH model. New Phytol..

[24] Seleznyova AN, Hanan J (2018). Mechanistic modelling of coupled phloem/xylem transport for L-systems: Combining analytical and computational methods. Ann. Bot..

[25] Prusinkiewicz P (2009). Control of bud activation by an auxin transport switch. PNAS.

[26] Renton M, Hanan J, Ferguson BJ, Beveridge CA (2012). Models of long-distance transport: How is carrier-dependent auxin transport regulated in the stem?. New Phytol..

[27] Pellerin BP, Buszard D, Georgallas A, Nowakowski RJ (2012). A novel framework to consider endogenous hormonal control of apple tree flowering. HortScience.

[28] Pallas, B., Costes, E. & Hanan, J. Modeling bi-directional signals in complex branching structure: Application to the control of floral induction in apple trees. In *Proceedings—2016 IEEE International Conference on Functional-Structural Plant Growth Modeling, Simulation, Visualization and Applications*, 150–157 (2016).

[29] Reyes F (2019). MuSCA: A multi-scale source-sink carbon allocation model to explore carbon allocation in plants. An application on static apple-tree structures. Ann. Bot..

[30] Pradal C, Dufour-Kowalski S, Boudon F, Fournier C, Godin C (2008). OpenAlea: A visual programming and component-based software platform for plant modelling. Funct. Plant Biol..

[31] Godin C, Costes E, Sinoquet H (1999). A method for describing plant architecture which integrates topology and geometry. Ann. Bot..

[32] Foster T, Johnston R, Seleznyova A (2003). A morphological and quantitative characterization of early floral development in apple (Malus × domestica Borkh.). Ann. Bot..

[33] Wünsche J-N, Palmer JW, Greer DH (2000). Effects of crop load on fruiting and gas-exchange characteristics of ’Braeburn’/M.26 apple trees at full canopy. J. Am. Soc. Hortic. Sci..

[34] Sonohat G, Sinoquet H, Kulandaivelu V, Combes D, Lescourret F (2006). Three-dimensional reconstruction of partially 3D-digitized peach tree canopies. Tree Physiol..

[35] Wünsche J-N, Ferguson IB (2005). Crop load interactions in apple. Hortic. Rev..

[36] Costes E (2008). MAppleT: Simulation of apple tree development using mixed stochastic and biomechanical models. Funct. Plant Biol..

[37] Costes E (2014). Bud structure, position and fate generate various branching patterns along shoots of closely related Rosaceae species: a review. Front. Plant Sci..

[38] Renton M, Guédon Y, Godin C, Costes E (2006). Similarities and gradients in growth unit branching patterns during ontogeny in ‘Fuji’ apple trees: A stochastic approach. J. Exp. Bot..

[39] Migault V, Pallas B, Costes E (2017). Combining genome-wide information with a functional structural plant model to simulate 1-year-old apple tree architecture. Front. Plant Sci..

[40] Crabbé JJ (1984). Vegetative vigor control over location and fate of flower buds, in fruit trees. Acta Hortic..

[41] Lauri P-E, Terouanne E (1998). The influence of shoot growth on the pattern of axillary development on the long shoots of young apple trees (Malus domestica Borkh.). Int. J. Plant Sci..

[42] Wolters H, Jürgens G (2009). Survival of the flexible: Hormonal growth control and adaptation in plant development. Nat. Rev. Genet..

[43] Freiman A (2015). Expression of flowering locus T2 transgene from *Pyrus communis* L. delays dormancy and leaf senescence in Malus × domestica Borkh, and causes early flowering in tobacco. Plant Sci..

[44] Putterill J, Varkonyi-Gasic E (2016). FT and florigen long-distance flowering control in plants. Curr. Opin. Plant Biol..

[45] Wahl V (2013). Regulation of flowering by trehalose-6-phosphate signaling in *Arabidopsis thaliana*. Science.

[46] Regnault T (2015). The gibberellin precursor GA12 acts as a long-distance growth signal in Arabidopsis. Nat. Plants.

[47] Binenbaum J, Weinstain R, Shani E (2018). Gibberellin localization and transport in plants. Trends Plant Sci..

[48] Willaume M, Lauri P-E, Sinoquet H (2004). Light interception in apple trees influenced by canopy architecture manipulation. Trees Struct. Funct..

[49] Lauri P-E, Lespinasse J-M (1993). The relationship between cultivar fruiting-type and fruiting branch characteristics in apple trees. Acta Hortic..

[50] Segura V, Durel C-E, Costes E (2009). Dissecting apple tree architecture into genetic, ontogenetic and environmental effects: QTL mapping. Tree Genet. Genomes.

[51] Celton J-M (2014). Fruit self-thinning: A trait to consider for genetic improvement of apple tree. PLoS ONE.

[52] Mouradov A, Cremer F, Coupland G (2002). Control of flowering time: Interacting pathways as a basis for diversity. Plant Cell.

[53] Lee J, Lee I (2010). Regulation and function of SOC1, a flowering pathway integrator. J. Exp. Bot..

[54] Bernier G, Havelange A, Houssa C, Petitjean A, Lejeune P (1993). Physiological signals that induce flowering. Plant Cell.

[55] Aki T, Shigyo M, Nakano R, Yoneyama T, Yanagisawa S (2008). Nano scale proteomics revealed the presence of regulatory proteins including three FT-like proteins in phloem and xylem saps from rice. Plant Cell Physiol..

