# Supplementary material for: Modelling transport of inhibiting and activating signals and their combined effects on floral induction: application to apple tree
Source: Sci Rep. 2020 Aug 4;10:13085. doi: 10.1038/s41598-020-69861-8 (PMC7403595; doi:10.1038/s41598-020-69861-8)
Supplement: Supplementary file 1 — Supplementary Information. [file 41598_2020_69861_MOESM1_ESM.docx]

Supplementary information

**Modelling transport of inhibiting and activating signals and their combined effects on floral induction: application to apple tree**

Farés Belhassine, Damien Fumey, Jérôme Chopard, Christophe Pradal, Sébastien Martinez, Evelyne Costes, Benoît Pallas

The following supplementary information is available for this article:

**Methods S1.** Description of the allometric relationships used for individual leaf area reconstruction

**Fig. S1** MTG and architecture representation in the modelling approach

**Fig. S2** 3D representation of the leaf and fruit removal treatments

**Fig. S3** Simulation results on simplified structures (6 shoots) with leaf removal.

**Fig. S4** Simulation results on simplified structures (2 branches) with leaf removal.

**Fig. S5** Simulation results on simplified structures (4 branches) with fruit and leaf removal and assuming a “limiting factor” approach.

**Fig. S6** Representation of the functions used for signal transport and meristem sensitivity after calibration.

**Fig. S7** Distribution of individual shoot leaf area for the input architectures.

**Methods S1.** Description of the allometric relationships used for individual leaf area reconstruction

Measurements were performed on 60 short shoots (=< 5cm) and 60 long shoots (>5cm) to estimate leaf area number, values and distribution along each shoot. The following allometric relationships were thus established:

For the number of leaves (*N_L_*) for each shoot type:

$N_{L}=42.8 \times l+4.24$

With l the length (m) of each shoot computed from 3D coordinates of the base and top of the shoots. (R²=0.52)

Individual leaf areas (*L_ai_*) along each shoot were computed as the product between the maximal individual leaf area (*L_M_*) depending on shoot type and a relative leaf area ranging between 0 and 1 (*R_l_*) and used to represent the leaf area profile along shoots, as follows:

$L_{ai}=R_{l}\times L_{M}$

Based on measurements *L_M_* was set to 38.6 and 27.6 cm² for short and long shoots, respectively.

*R_l_* was computed from a quadratic equation depending on the relative rank of the leaf (*r_i_*) along shoots with *r_i_* defined as the ratio of leaf insertion rank to shoot leaf number (*N_L_*)

For short shoots, $R_{l}=-1.03\times R_{l}^{2}+1.8\times R_{l}+0.15$ (R²=0.95)

For long shoots, $R_{l}=-0.93\times R_{l}^{2}+0.87\times R_{l}+0.80$ (R²=0.96)


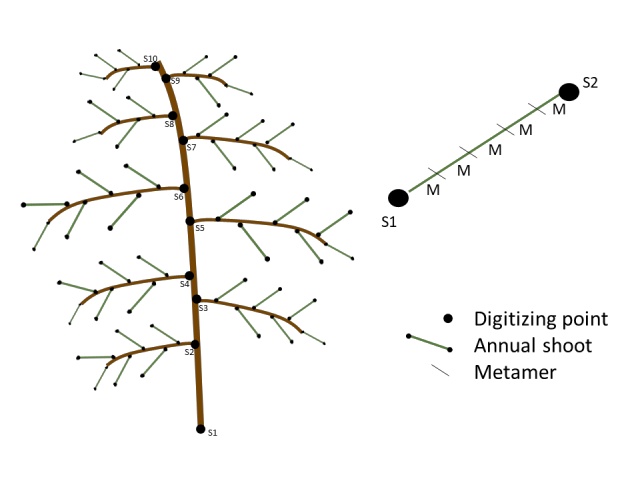


**Fig. S1** MTG structure used in the model with three scales (tree, stem segment and metamer). The green segments represents annual shoots. M represent metamers considered in this MTG for the most recent shoots, only. Black points refer to the different digitizing points on the trees which are used to separate each stem segment.


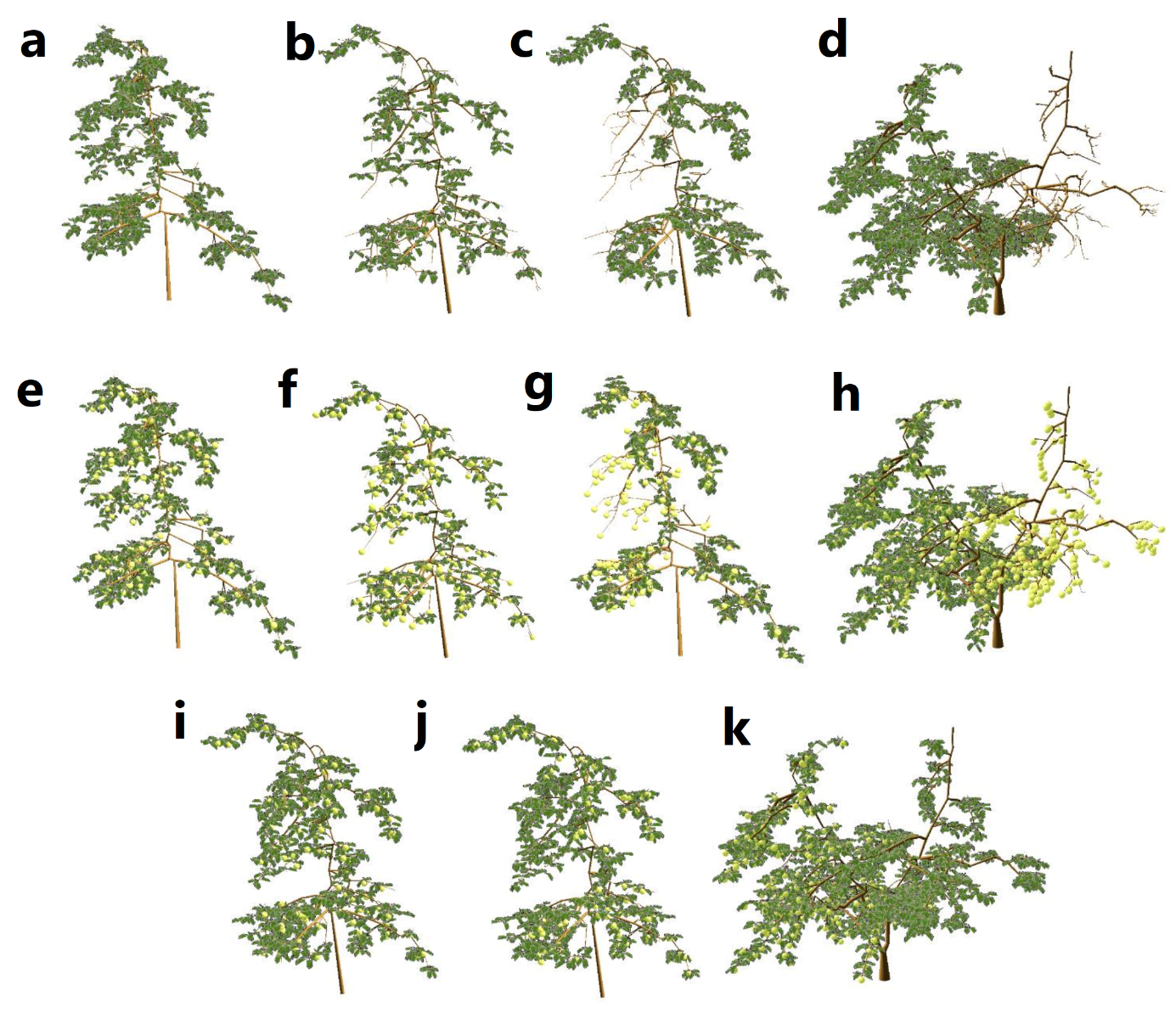


**Fig. S2** 3D visualization of tree structures used for calibration (a,b,c,d,e,I,j,k) and validation (f,g,h) of the model’s parameters. (a) is the representation of control trees in OFF conditions. (b), (c) and (d) defoliated OFF trees at the shoot, branch and half-tree (Y-Shape tree) scales, respectively. (e) control trees in ON conditions. (f), (g) and (h) defoliated ON trees at the shoot, branch and half-tree scale, respectively. (i), (j) and (k) defruited ON trees at the shoot, branch and half-tree scale, respectively.


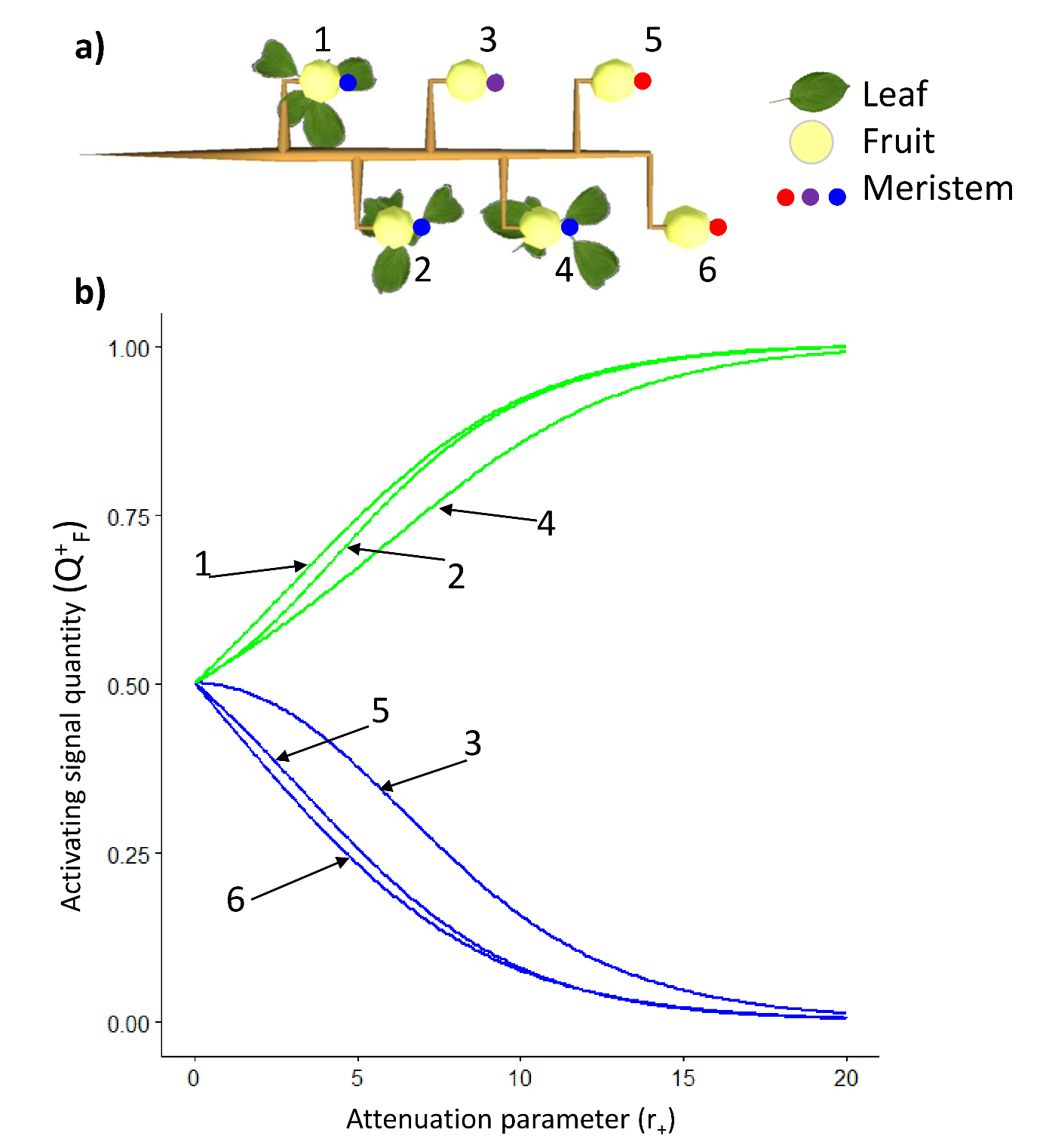


**Fig. S3** Activating signal concentration (*Q^+^_F_*) in six meristems for different values of the signal ‘attenuation’ parameter (*r_+_*) (b). Simulations were performed on a hypothetical structure composed of six shoots, half of them leafy, each shoot being located at 15 cm from each other (a). 1, 2 and 4 are leafy shoots and 3, 5 and 6 are non-leafy shoots.


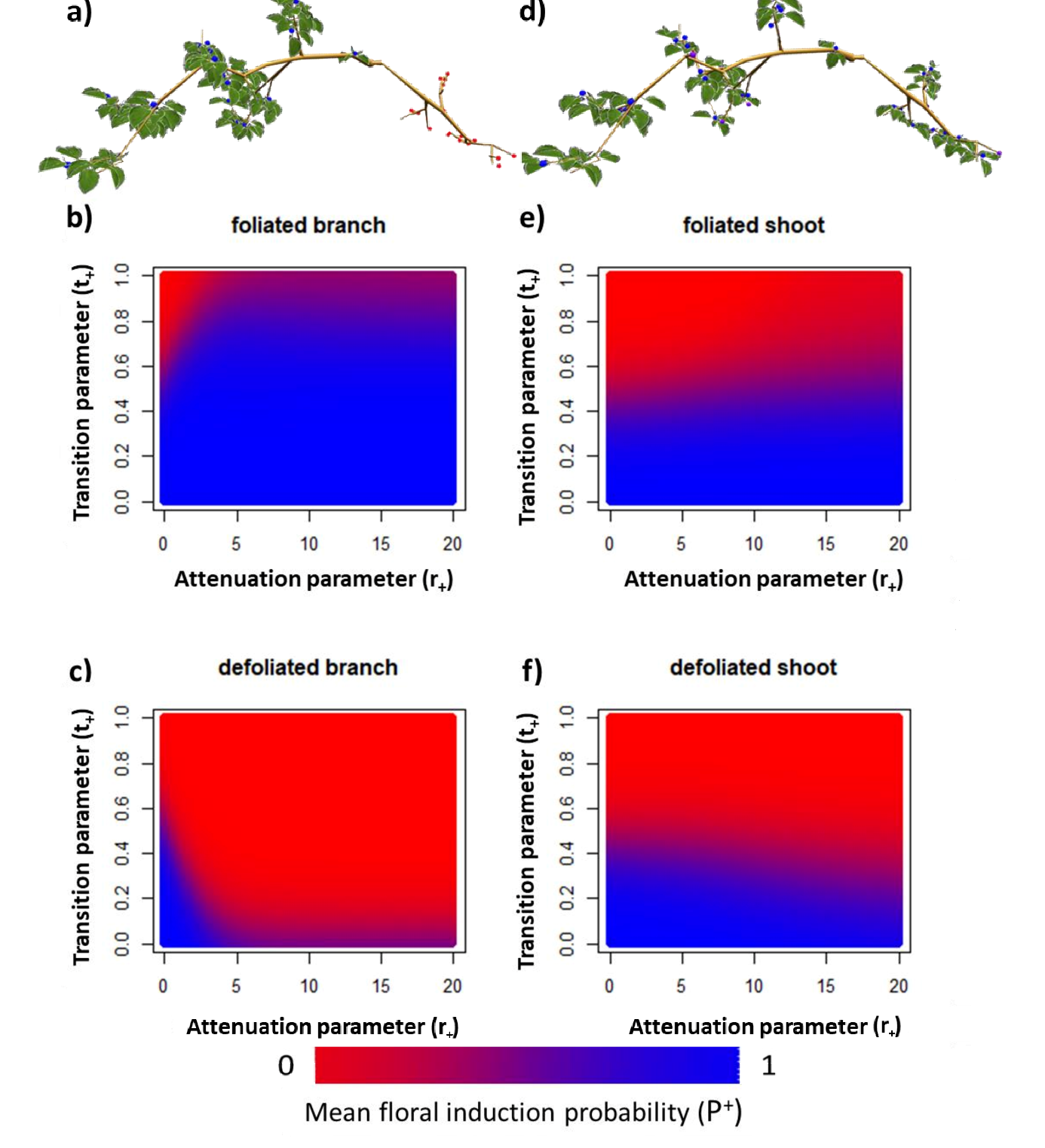


**Fig. S4** Mean floral induction probability (*P^+^*) depending on the quantity of activating signal produced by leaves for different values of the signal ‘attenuation’ (r_+_) (200 values) and ‘transition’ parameters (t_+_) (100 values). Simulations were performed on two hypothetical structures composed of two branchesfwith one leafy and one non-leafy branch (a) and two branches with homogeneous leaf removal on half of the shoots (d). (b) and (c) represent the mean floral induction proportion in leafy and non-leafy branch, respectively for the structure represented in (a). (e) and (f) represent the mean floral induction proportion in leafy and non-leafy shoots, respectively for the structure represented in (d). Simulations were performed assuming a shape parameter value (v_+_) equal to 0.25.


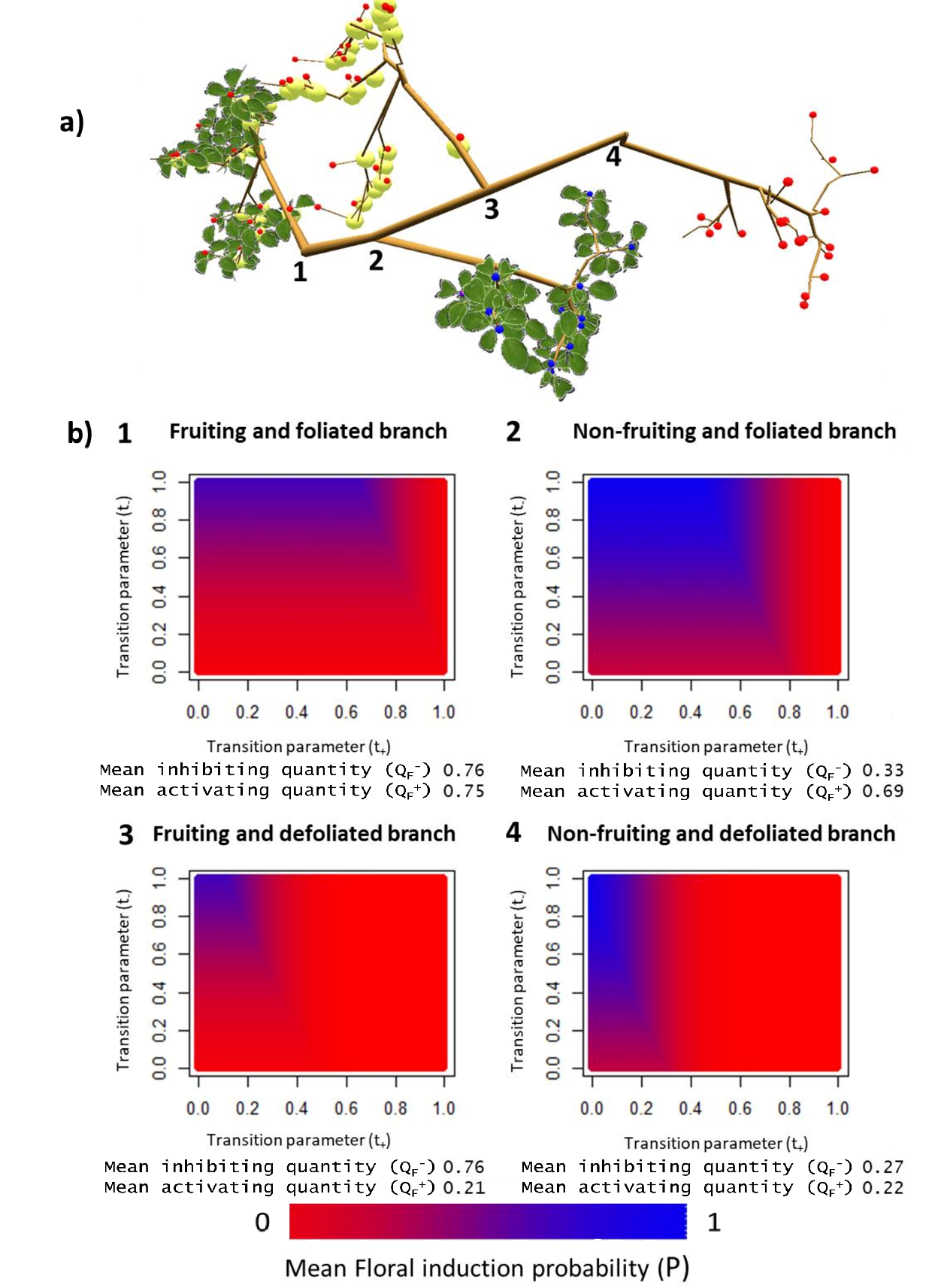


**Fig. S5** Mean floral induction probability (*P*) in shoot apical meristem for different ‘transition’ parameter values for the activating (*t_+_*) and inhibiting (*t_-_*) signals. Floral induction proportions were computed on a hypothetical structure composed of one fruiting and leafy branch (1), one non-fruiting and leafy branch (2), one fruiting and non-leafy branch (3) and one non-fruiting and non-leafy branch (4) (a). (b) represent the mean floral induction proportion in the different branches. Mean inhibiting and mean activating above the heatmaps represent the mean of the inhibiting and activating signals quantities for all meristems in each branch. Simulations were performed with the limiting-factor formalism to account for the effect of the inhibiting and activating signal on floral induction. In these simulations ‘shape’ parameters (*v_-_*, *v_+_*) equal to 0.25 and ‘attenuation’ parameters (*r_+_*, *r_-_*) equal to 2.5 were used.

**
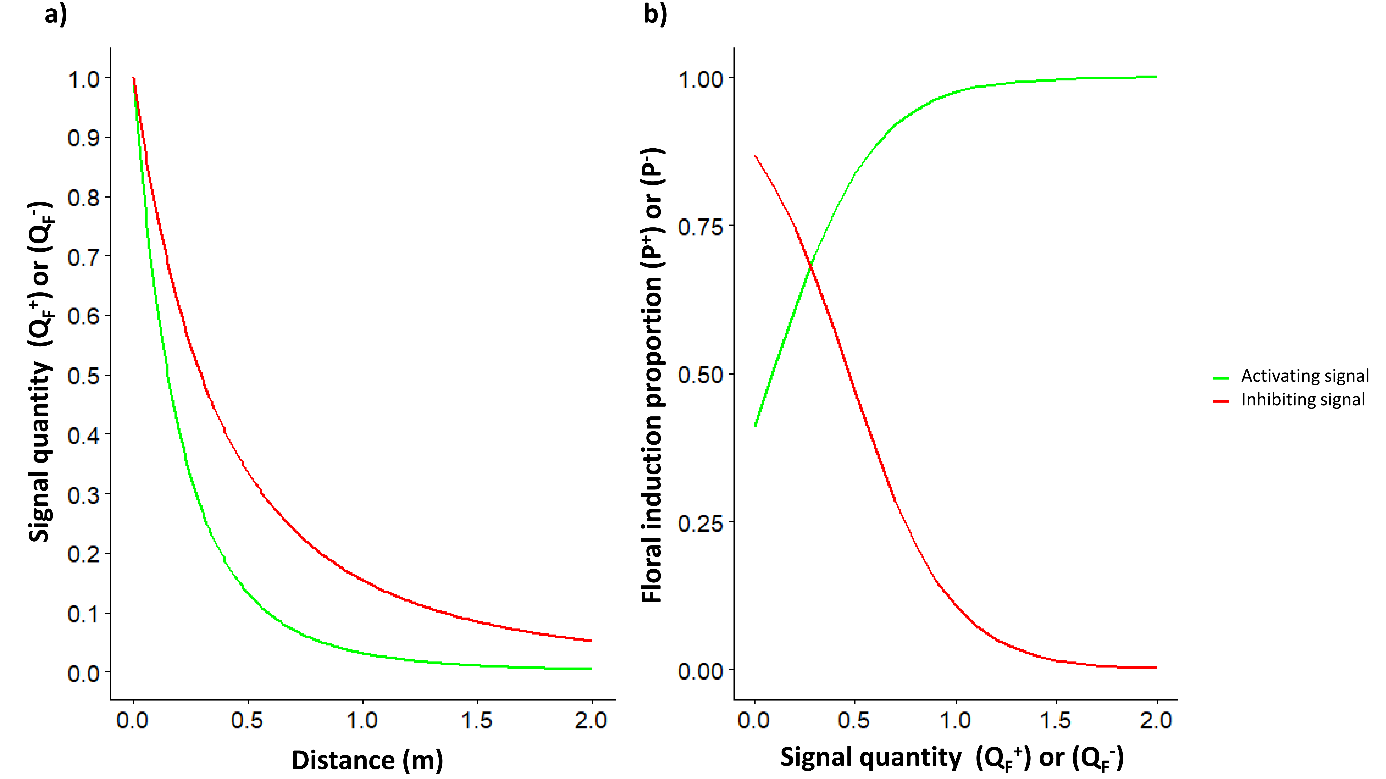
**

**Fig. S6** (a) Representation of equations for activating (green) and inhibiting (red) signal quantity (*Q^+^_F_* and *Q^-^_F_*) depending on distances from the emitting sources, with the values of ‘attenuation’ parameters (r_+_= 5 and r_-_ = 2.7) estimated after model calibration. (b) Representation of floral induction proportion for different activating (green) and inhibiting (red) signal quantity (*Q^+^_F_* and *Q^-^_F_*), with ‘transition’ and ‘shape’ parameter values (t_+_ = 0.09, v_+_ = 0.25 and t_-_ = 0.47, v- = 0.25) estimated after model calibration.


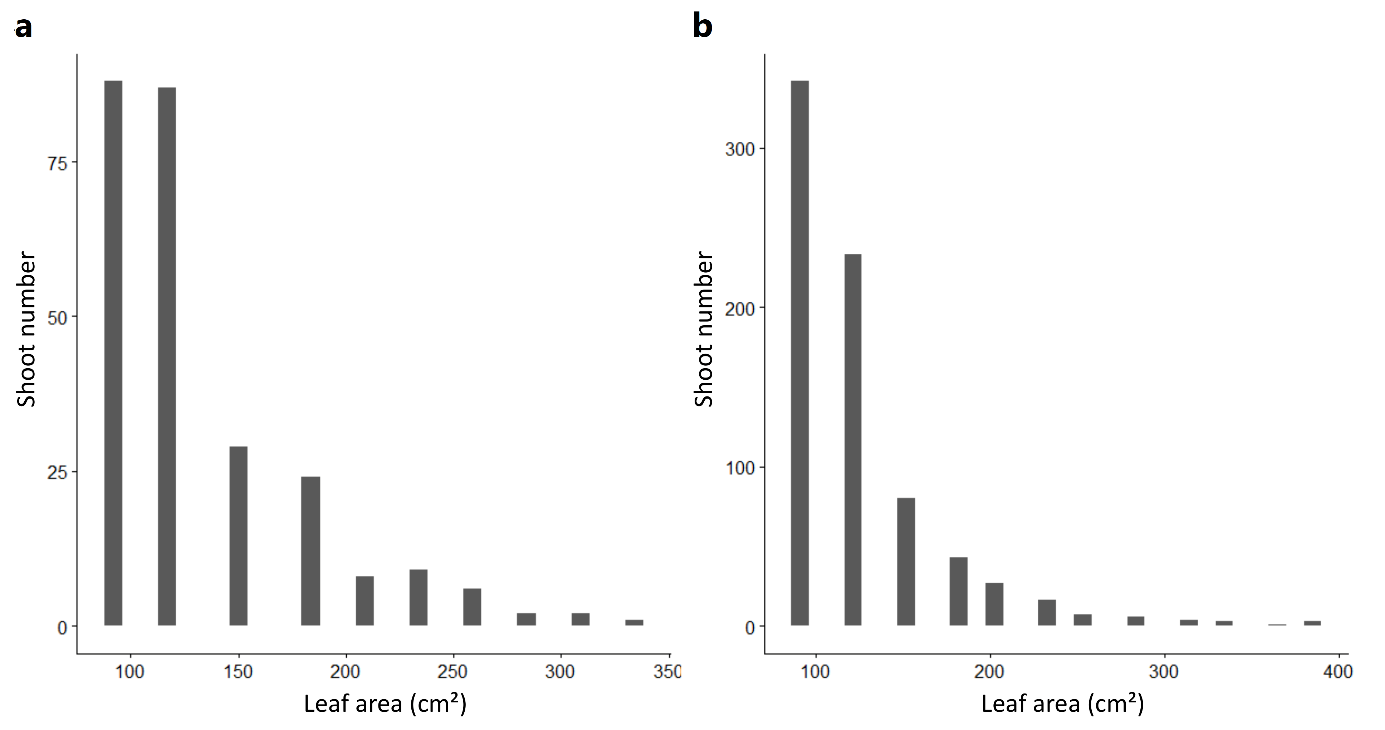


**Fig. S7** Distribution of shoot leaf areas for the tree structures used for simulations with one main axis (Solaxe, a) and two main axes (Y-shape, b).
